# Mitochondrial genomes and comparative genomics of *Aphanomyces astaci* and *Aphanomyces invadans*

**DOI:** 10.1038/srep36089

**Published:** 2016-11-03

**Authors:** Jenny Makkonen, Arto Vesterbacka, Frank Martin, Japo Jussila, Javier Diéguez-Uribeondo, Raine Kortet, Harri Kokko

**Affiliations:** 1 Department of Environmental and Biological Sciences, University of Eastern Finland, P.O. Box 1627, FI-70211 Kuopio, Finland; 2 United States Department of Agriculture, ARS, 1636 E. Alisal St., CA-93905 Salinas, USA; 3 Real Jardín Botánico CSIC, Plaza de Murillo 2, E-28014 Madrid, Spain; 4 Department of Environmental and Biological Sciences, University of Eastern Finland, P.O. Box 111, FI-80101 Joensuu, Finland; 5 Department of Biology, Biological & Geological Sciences, University of Western Ontario, 1151 Richmond St N, London, Ontario N6A 5B7, Canada

## Abstract

The genus *Aphanomyces* (Saprolegniales, Oomycetes) includes species with a variety of ecologies from saprotrophs to plant and animal parasites. Two important species in this genus are *A. astaci,* the cause of crayfish plague and its close relative, *A. invadans*, which causes the epizootic ulcerative syndrome on fish. In this study, we have assembled and annotated the mitochondrial (mt) genomes of *A. astaci* and *A. invadans* from the whole genome shotgun sequence reads (PRJNA187372; PRJNA258292, respectively). The assembly was generated from *A. astaci* Pc-genotype strain APO3 and *A. invadans* strain NJM9701. The sizes of the mtDNAs were 49,489 bp and 49,061 bp for *A. astaci* and *A. invadans,* respectively. The species shared similar genetic content and organization encoding 35 proteins, two ribosomal RNAs, three putative open reading frames and 33 transfer RNAs of 19 amino acids for peptide synthesis. Both species also had a large inverted repeat region (LIR) of approximately 12 kb, the LIR contained large and small ribosomal RNAs and eight protein coding genes. These annotated mt genomes serve as a valuable genetic backbone for further development of diagnostic methods and phylogenetic and migration studies of the animal parasitic species of *Aphanomyces*.

Oomycetes (Stramenopiles) include a variety of species, both saprotrophic and pathogenic, having plant and animal hosts in terrestrial and aquatic ecosystems^1^. The plant pathogenic Oomycetes (Peronosporoales) have been reported to cause economically and culturally significant global epidemics, such as potato late blight (*Phytophthora infestans*) and sudden oak death (*Phytophthora ramorum*), thus having impacted both human society and natural ecosystems^2^. Aquatic clades of Oomycetes, like Saprolegniales, are a diverse group adapted to several hosts and different life styles in aquatic environment^3^. Some species of this aquatic clade impose notable ecological and economic impacts on aquaculture and fisheries^4^,^5^. The genus *Aphanomyces* (Saprolegniales) includes a variety of species belonging to plant pathogens, animal parasites and saprotrophs^5^, two important animal parasitic species are *A. astaci*^6^, the causative agent of crayfish plague, and *A. invadans*^7^, which causes epizootic ulcerative syndrome (EUS) in both wild and farmed fish^8^.

*Aphanomyces astaci* is a highly specialized parasite of freshwater crayfish species originating from the North American continent^9^,^10^. When it spread into European aquatic ecosystems via Italy after the 1860’s, *A. astaci* became a devastating pathogen for native European crayfish species^6^,^11^. The disease has since then dramatically altered the balance of European aquatic ecosystems^12^. To date, five *A. astaci* genotypes have been detected in Europe, each of them being introduced with a different North American crayfish species^5^,^13^,^14^,^15^. However, the true spectrum of *A. astaci* host species may be much wider, since a limited amount of information is available about genotypes that may infect other North American crayfish species^16^. The presumably rapid co-evolution of *A. astaci* and its native European crayfish hosts^17^, combined with its fairly simple life-history, makes it especially interesting model organism for host parasite co-evolution studies.

*Aphanomyces invadans*, formerly known as *Aphanomyces piscisida,* causes epizootic ulcerative syndrome (EUS) in both wild and farmed fish^8^. More than 100 fish species have been reported to be affected by EUS^18^. The disease was first detected in Japan^19^, but has been subsequently reported from continental Asia, Australia, North America and Africa, with different names being given^20^. Based on several molecular studies, a single clone of *A. invadans* has been broadly distributed worldwide^21^,^22^. *A. astaci* and *A. invadans* seem to be closely related on the genetic level^5^, and they also share several common life style characteristics; *i.e.* lack of a sexual reproduction cycle, repeated zoospore emergence, and highly specialized parasitic nature, (an alternative host for either species from different taxonomic class is currently unknown)^5^,^21^,^23^.

In Oomycetes, mitochondrial DNA (mtDNA) is maternally inherited^24^ and has been widely used for tracking the origin and geographic migration of a broad variety of species, perhaps most interestingly studying the historic migration of *Phytophthora infestans* using herbarium specimens^25^,^26^,^27^. Mitochondrial (Mt) genomes of several genera of Oomycetes have recently been characterized and their size and organization have been found to be highly variable, from approximately 37 kb for *Phytophthora infestans*^28^ to 60 kb for *Pythium ultimum*^29^. Currently, there are 15 Oomycete mt genomes published^30^,^31^,^32^,^33^,^34^,^35^,^36^,^37^, or available in sequence databases (KT946598), but with one exception these represent plant parasitic and saprophytic species while the animal pathogenic group has thus far been underrepresented.

The rapid development of sequencing techniques has offered new possibilities for detailed and comprehensive analyses of different organisms. Recently, *A. astaci* and *A. invadans* genomes were sequenced^38^. The present study describes the assembled mt genomes of these two closely related, animal pathogenic oomycetes, and are the first mt genomes representing the genus *Aphanomyces*. The presented results will form a solid basis that enables further studies of evolution, diversification and migration of *A. astaci* and *A. invadans*, and in a wider scale, the evolution of the animal parasitic lineages of Oomycetes.

## Results

### Genome size and organization

**Figure 1:**
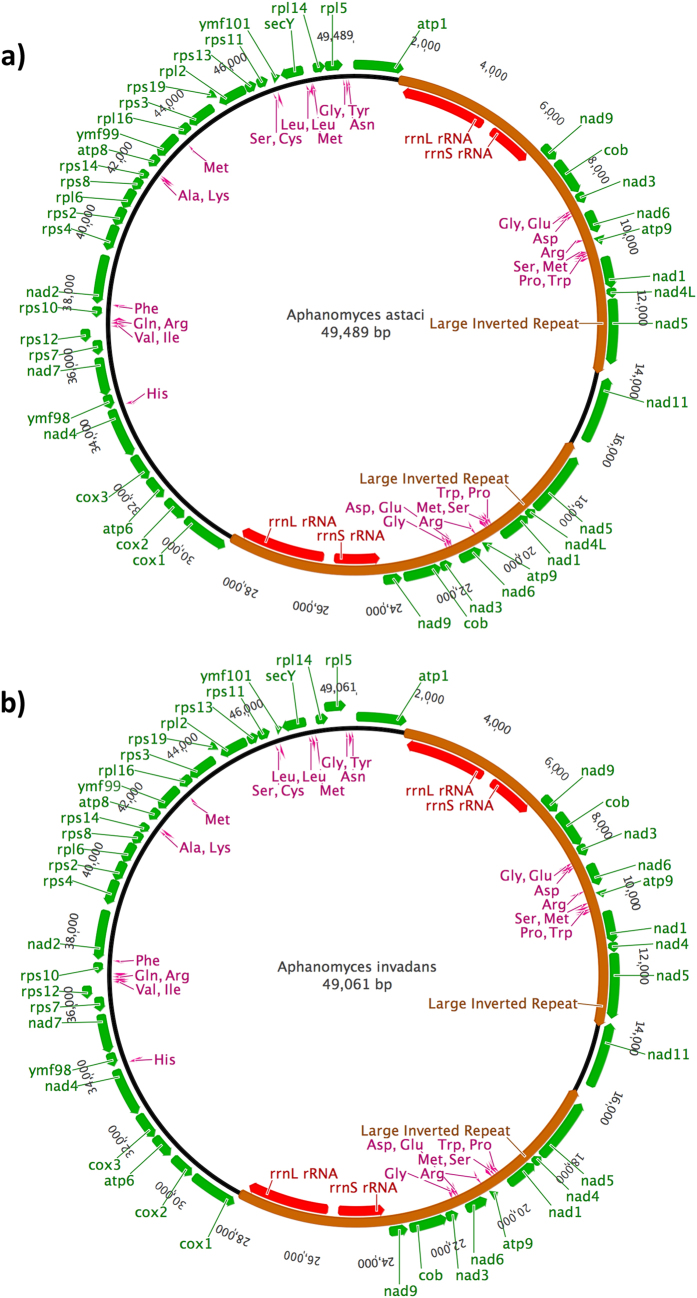
The organization of the mitochondrial genome including protein coding genes (green), large inverted repeat region (brown), ribosomal RNAs (red) and tRNAs (pink). (**A**) *Aphanomyces astaci* mtDNA (49,489 bp) and (**B**) *Aphanomyces invadans* mtDNA (49,061 bp).

The mt genome size of *A. astaci* was 49, 489 bp (mean coverage depth in the assembly of 525 reads) (Fig. 1A) while the *A. invadans* mt genome was 49,061 bp (mean coverage depth in the assembly of 520 reads) (Fig. 1B). The mtDNA content and orientation was similar in both species. There were large inverted repeats (LIRs) in the genomes of these species. In *A. astaci* there was a 12,570 bp LIR while in *A. invadans* there was a 12,366 bp LIR. The LIRs in these species represent approximately 50% of the mt genome size. Between the LIRs there were single copy regions of 2,342 bp in *A. astaci* and 2,133 bp in *A. invadans*, which coded for the *nad*11 gene. There was also a 22,008 bp single copy region in *A. astaci* and a 22,213 bp region in *A. invadans* that encoded 26 genes and three putative open reading frames (ORFs). A unique feature observed in both *Aphanomyces* mtDNAs was that the large (*rrnL*) and small (*rrnS*) ribosomal RNA subunits, located in the LIR, were concatenated and having a tail-to-tail orientation.

### Genome composition

The *A. astaci* mt genome was comprised of tightly packed coding regions (92.5%) with 60% of the intergenic spacer regions less than 20 bp. In contrast 32.0% of the intergenic regions in *A. invadans* were larger than 100 bp, while only 30% was less than 20 bp. The overall GC content of *A. astaci* mt genome was 22.6% with 23.9% GC content in coding regions and only 7.9% in intergenic regions. The mt genome of *A. invadans* was composed of 89.6% coding regions and 10.4% intergenic spacer regions. Overall GC content was 21.8% and the GC-content of coding regions was 22.2%, while for non-coding regions it was 15.8%. The largest intergenic spacer in both *A. astaci* (575 bp) and *A. invadans* (570 bp) was between *nad*5 in the second LIR-region and *nad*11 in the single copy region (Fig. 1A,B). The second largest intergenic spacer region of *A. astaci* (444 bp) was between *nad*5 in the first LIR-region and *nad*11, but for *A invadans* this spacer was only 129 bp and was completely located in the first LIR-region (Fig. 1B). In general, intergenic spacer regions were larger in the LIR-regions and the genes in the 22,213 bp single copy region were more tightly packed. In both species, the protein encoding genes had an ATG start codon and TAA stop codon. In both species, there were three exceptions for the stop codon for *rpl*2, *rpl*5 and *ymf*98 which were TAG not TAA. No introns were detected in the mt genome of either species.

### Protein coding genes and tRNAs

Altogether, the mtDNA of the studied *Aphanomyces* species encoded 35 proteins and two ribosomal RNAs. The gene set contained 18 respiratory chain proteins, 16 ribosomal proteins and an import protein *sec*Y (Table 1). Moreover, three putative ORFs for *ymf*98 (*orf*147), *ymf*99 (*orf*217) and *ymf*101 (*orf*64) were observed. These ORFs have also been observed in other oomycetes. The LIR-region of both species encoded eight genes (*atp*9*, cob, nad*1*, nad*3, *nad*4L, *nad*5, *nad*6 and *nad*9) and the ribosomal RNAs *rrnL* and *rrnS*. The single copy -region of 22,008 bp on the opposite side of the LIRs encoded 29 genes, containing all of the ribosomal proteins, a part of complex I and all of the complex IV and V respiratory chain proteins, and *sec*Y import protein (Table 1, Fig. 1).

In the mt genome of both species, there was an identical set of 33 tRNAs for 19 different amino acids. No tRNA encoding amino acid threonine were detected. The sizes of the tRNAs varied between 71 and 89 bp and the GC content between 27.6 and 50.0%. Eight tRNAs were located in LIR-region (Table 1, Fig. 1).

### Phylogenetic comparisons

The comparison of *A. astaci* and *A. invandas* mtDNAs indicated that these two closely related species had an identical mitochondrial gene order and orientation. The similarity percentage of the complete mt genome was 91.6%, being highest (93.4%) in the coding regions, and the lowest (66.7%) in the intergenic spacer regions. The pairwise identity between the LIR of each species was 91.6%, identical to comparisons of the complete mt genome. The individual gene showing the greatest level of similarity was *rrnS* rRNA, where the similarity reached 97.8%. The highest divergence was observed in the 444 bp intergenic spacer region between the genes *nad*11 and *nad*5, locating in the end of the LIR, where the similarity was only 23.4%.

**Figure 2:**
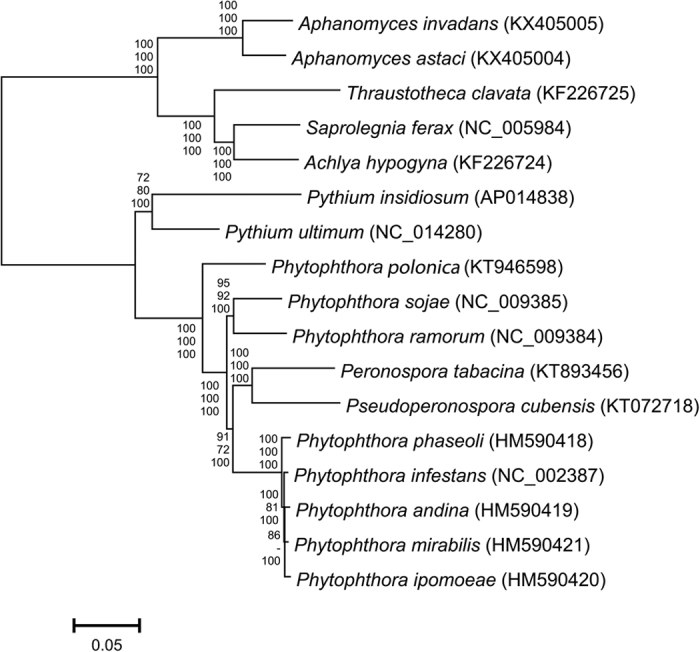
Maximum likelihood (ML) tree of *Aphanomyces astaci, A. invandans* and 15 other oomycetes based on concatenated mitochondrial gene set of 37 genes. Values at the nodes represent bootstrap support for maximum likelihood (top), maximum parsimony (middle) and posterior probabilities for Bayesian (bottom).

**Figure 3:**
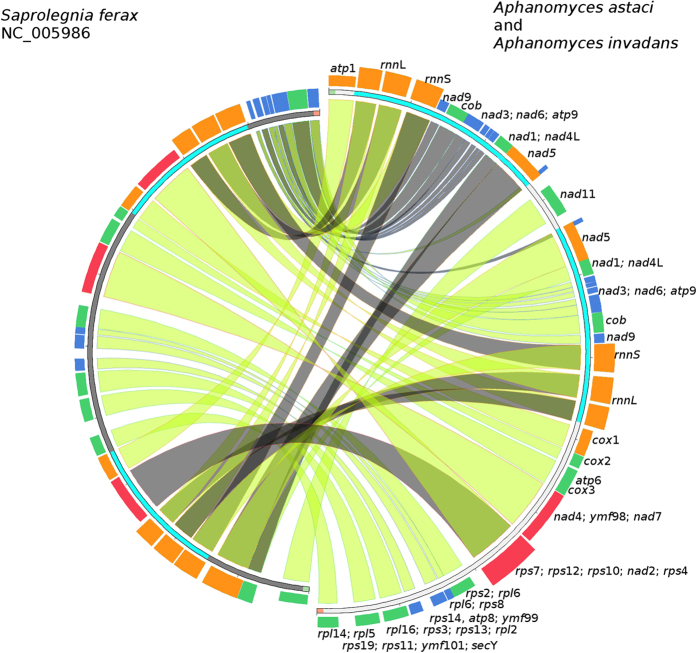
Structural comparison of *Aphanomyces astaci* and *A. invadans* mtDNA against *Saprolegnia ferax*. Green and red block in the beginning and end of the sequences indicate the sequence orientation. The inverted repeats are marked with turquoise color. The colored blocks outside the sequence describe the blast hit scoring, best quartile being red, then orange, green and blue, respectively. The connective lines are green, when the sequences have inverted orientation and grey, when coded on same strand.

A phylogenetic comparison of a concatenated gene set (n = 37) of 17 oomycete mt genomes placed the *Aphanomyces* species into the Saprolegnian clade (Fig. 2). Furthermore, the genetic structure of *A. astaci* and *A. invadans* mt genomes were compared against *S. ferax* (Fig. 3), which is their most closely related species in the Saprolegniaceae with a mt genome currently available. The structural comparisons showed that in the approximately 22 kb single copy region, there was a conserved 25 gene order with the exception that in *Aphanomyces* the *nad*2*-rps*10*-rps*12*-rps*7 genes were inserted between *rps*4 and *nad*7. While the *rnnS* and *rnnL* were encoded in the LIR in both genera, in *Aphanomyces* spp. they had a tail-to-tail-positioning. The remaining list of the LIR encoded genes, containing 8 and 4 additional genes for *Aphanomyces* spp. and *S. ferax,* respectively, was completely different. The greatest sequence similarity between the genera were the two approximately 4 kb blocks, containing *rps*7, -12, -10, *nad*2 and *rps*4 genes, and *nad*4, *ymf*98 and *nad*7 -genes (Fig. 3). Overall, the sequence identities of the gene blocks varied between 83 and 95%.

## Discussion

Here we assembled and annotated the mt genomes of *A. astaci* and *A. invadans,* which both represent a parasitic lineage of the genus *Aphanomyces* in the Saprolegniales lineage of the oomycete family. The species shared similar genetic content and organization and both species also had a large inverted repeat region (LIR) of approximately 12 kb, which contained large and small ribosomal subunits and eight protein coding genes.

The sizes of the *A. astaci* and *A. invadans* mt genomes (49,489 bp and 49,061 bp, respectively), are among the larger range of mtDNA sizes that have been observed in the oomycetes. The largest mt genome so far has been reported for two *Pythium* species, *P. ultimum*^29^ and *P. insidiosum*^36^, having a mt genome size of 59.7 and 55 kb, respectively (Table 2). A more closely related species to *Aphanomyces*, *Saprolegnia ferax,* had a mtDNA genome of approximately 47 kb^30^, being similar to the genome size found for *Achlya hypogyna* and *Thraustotheca clavata*^32^. In contrast, *Phytophthora* species and *Peronospora tabacina* do not have large inverted repeats, resulting in generally smaller mt genomes ranging from approximately 37 to 43 kb^31^,^33^,^34^. Comparison of the mt genomes of 17 oomycete taxa with and without inverted repeats indicates that genome sizes are fairly consistent when the second LIR copy is excluded (ranging from approximately 36 to 43 kb) with subsequent differences in genome size influenced by the numbers of putative ORFs that are present (Table 2). Also, the GC% of oomycete mtDNAs were uniform and generally low, from 19.0% of *P. ramorum* to 23.4% of *T. clavata,* with both *Aphanomyces* species coming between these.

The large inverted repeat (LIR) regions, observed in mt genomes of *A. astaci* and *A. invadans,* ranged in sizes from 12,570 bp to 12,366 bp, respectively. The smallest inverted repeats have been detected in *Phytophthora ramorum* (~1.1 kb)^31^, and the largest from *Pythium* species (~20 kb) (Table 2)^39^. LIR’s have been considered as the major contributor to the mt genome size variation^40^ as exemplified among the 17 oomycete taxa in Table 2. The role of these repeat regions have been thought to be the stabilization of the mtDNA structure^31^. No signs of the flip-flop isomerization, previously observed in oomycetes *Achlya ambisexualis*^41^, *A. klebsiena*^42^, *S. ferax*^30^ and *P. ramorum*^43^, were detected in the above noted *Aphanomyces* species.

The mt genomes of *A. astaci* and *A. invadans* encoded 35 protein coding genes and three putative ORFs. In addition, two ribosomal RNAs and 33 tRNAs for 19 amino acids, were detected. In comparison to other sequenced aquatic oomycete species^32^, the genetic composition, number of genes, and GC% of the mtDNA observed was similar. The largest structural difference was that in both *Aphanomyces* species, a large part of *nad*-genes (Table 1, Fig. 3) were located in LIR-region. In addition, the set of putative ORFs was different; *ymf*98 (*orf*147), *ymf*99 (*orf*217) and *ymf*101 (*orf*64) were found in both *Aphanomyces* spp. with *ymf*98 and *ymf*101 shared with *S. ferax* while *ymf*99 was not. Furthermore, *S. ferax* also had unique putative *orf*’s −273 and −312, which were not present in the studied *Aphanomyces* mt genomes. Similarities were also observed in the single copy region between arms of the LIR with the same gene order between both genera with the exception that the *Aphanomyces* spp. had 4 genes and 5 tRNAs inserted between *rps4* and *nad7*. The gene order between *secY* and *rps4* (15 genes, 5 tRNAs) is highly conserved and also observed in the Peronosporales taxa^29^,^31^,^33^.

Based on the phylogenetic analyses, *A. astaci* and *A. invadans* group within the Saprolegnian lineage of Oomycetes (Fig. 2), the results are in agreement with previous studies utilizing nuclear molecular markers^2^,^5^,^44^. Furthermore, our unpublished results show a high intraspecific variation in the mt genomes of different *A. astaci* genotypes, the significance of which awaits additional analysis. Surprisingly the two downy mildews included in this analysis (*Pseudoperonospora cubensis* and *Peronospora tabacina*) were grouped within the *Phytophthora* clade, indicating additional analysis with a larger number of species is needed to clarify this phylogenetic relationship. Mitochondrial barcoding genes have been shown to be a valuable addition to the taxonomic resources of the oomycetes^45^. Especially in *Phytophthora*, mitochondrial variation, exhibiting both inter- and intraspecific variation^46^, has previously been used to trace the spread of the different mitochondrial haplotypes^47^,^48^, and as a marker for examining the population structure of historical samples of *P. infestans*^25^,^26^,^27^.

The two *Aphanomyces* species examined herein are the first oomycete aquatic animal pathogens with fully assembled mtDNA genomes. The present results provide a useful genetic resource to further explore the population structure, evolution and origins of these devastating animal pathogenic oomycetes. The assembled mtDNA’s will serve as a future resource basis and reference sequence. When working with clonally reproducing organisms such as *A. astaci* and *A. invadans,* the mt genome likely provides a valuable marker for population studies.

## Material and Methods

### The strains

*Aphanomyces astaci* strain AP03^49^ was isolated from indigenous European crayfish, *Austropotamobius pallipes,* suffering of crayfish plague in La Garrotxa National Park in North-Eastern Pyrenees, Cataluña, Spain. The disease outbreak was caused by the Pc-(D)-genotype of *A. astaci*, which was transmitted to native *A. pallipes* population from the neighboring invasive red swamp crayfish (*Procambarus clarkii)* population^49^. The strain was isolated and maintained in Real Jardín Botánico, Consejo Superior de Investigaciones Científicas (CSIC), Spain.

*Aphanomyces invadans* strain NJM9701, originally isolated from Ayu (*Plecoglossus altivelis*) in Japan in 1997^50^ was the subject for the mt genome assembly and annotation in this study. The strain has been used as a starting material for the whole genome shotgun sequencing of *A. invadans* (PRJNA188085) and our study utilized the sequence data (SRS473776) produced in this sequencing project.

### DNA extraction

For genomic DNA isolation, the selected *A. astaci* strain was grown for three days at 24 °C in peptone glucose broth^51^. Briefly, fresh mycelia (1 g), were ground to a fine powder under liquid nitrogen, mixed with 10 mL of extraction buffer (0.2 M Tris HCl pH 8.5, 0.25 M NaCl, 25 mM EDTA, 0.5% SDS), 7 mL Tris-equilibrated phenol and 3 mL of chloroform:isoamyl alcohol (24:1), incubated at room temperature for 1 h and centrifuged at 6 000× g for 30 min. The aqueous phase was extracted with equal volume of chloroform:isoamyl alcohol (24:1) and centrifuged at 10 000 × g for 15 min. A total of 50 μl of 10 mg/mL RNase A was added to the aqueous phase and the tubes were incubated at 37 °C for 30 min. Then, isopropanol (0.6 volumes) was added, the samples were mixed gently, and the DNA was precipitated on ice for 30 min. DNA was collected by centrifugation at 10,000 g for 20 min, washed with 70% ethanol, dried and resuspended in DNase/RNase-free water. DNA was checked for quality and RNA contamination by gel electrophoresis using an 0.8% agarose gel.

### Library preparation and genome sequencing

*A. astaci* (PRJNA187372) whole genome shotgun sequencing was generated by BROAD Institute Illumina HiSeq2000^TM^ and the original raw reads (SRS473784 and SRS473776, respectively) were stored in the NCBI sequence read archive database. The whole genome shotgun sequencing of genomic DNA was generated by paired-end library via random selection, 3–5 kb jumping library and Fossil jumping library project (SRP018895). For *A. invadans*, the sequence reads were directly downloaded from the NCBI sequence read archive (PRJNA188085).

### Assembly and annotation

The generated reads were used to assemble the complete mt genome. One assembly of the WGS-reads of *A. astaci* was made using the RAY-assembler^52^. A scaffold representing a partial genome was generated and identified by BLASTn-search to match to the mt genome of *Saprolegnia ferax* (AY534144). To obtain a complete genome a *de novo* assembly was done with SeqMan NGen, version 4.1.2 (DNASTAR, Madison, WI, USA) with mitochondrial contigs identified by BLAST analysis with the *S. ferax* mt genome. The mitochondrial contigs were used in a templated assembly with all the sequencing reads with the resulting assembly evaluated for uniformity and depth of coverage. The contig was broken into smaller contigs when gaps/low coverage or inconsistences were observed and reassembled using the small templated assembly option of SeqMan NGen to extend the ends of the contigs and to close the gaps. The borders of the inverted repeat were identified when reviewing the assembly and noting a higher percentage of truncated reads at specific locations; additional sequence comparisons confirmed the border locations. Annotation of coding regions and prediction of ORFs was done with DS Gene v1.5 (Accelrys, San Diego, CA) using the universal genetic code with confirmation of gene identities done by BLAST analysis against published mt genome sequences from *Saprolegnia, Pythium*, and *Phytophthora* spp. Annotation of the tRNAs were conducted using tRNA Scan^53^. Assembly of the *A. invadans* mt genome followed the same procedure with SeqMan NGen. The sequences were submitted to NCBI GenBank with access numbers KX405004 (*A. astaci*) and KX405005 (*A. invadans*).

### Comparative genomics

A concatenated mt gene set (n = 37) of 17 oomycetes (Table 2) were included to phylogenetic comparisons. The genes included *atp*1, *atp*6, *atp*8, *atp*9, *cob*, *cox*1, *cox*2, *cox*3, *nad*1, *nad*11, *nad*2, *nad*3, *nad*4, *nad*4L, *nad*5, *nad*6, *nad*7, *nad*9, *rpl*14, *rpl*16, *rpl*2, *rpl*5, *rpl*6, *rps*10, *rps*11, *rps*12, *rps*13, *rps*14, *rps*19, *rps*2, *rps*3, *rps*4, *rps*7, *rps*8, *secY, rnnL*, and *rnnS* and the concatenation was made in Geneious 8.0^54^. The optimal substitution model, GTR + G, was selected with JModel Test in Topali v1.5^55^ and three types of phylogenetic trees were generated. The maximum likelihood tree was generated with PhyML^56^ with 100 bootstrap replicates. The Bayesian tree (MrBayes^57^) was conducted in two runs with 1 000 000 generations with sampling frequency of 20 and burn in 30%. The maximum parsimony (MP) analysis was done using PAUP version 4.0b10 (Sinaur Associates, Sunderland, MA) with a heuristic tree search with MULPARS on, steepest decent option off, random addition of sequences (10 replicates) and TBR branch swapping. To determine support for the various clades of the trees, the analysis was bootstrapped with 100 replicates. No outgroups were used as the sequence divergence (over 36% in several cases) and the amount of length mutations was too high.

The structural comparisons of aquatic oomycetes (*S. ferax, A. astaci* and *A. invadans*) mtDNAs were generated with Circoletto^58^, combining Blast search^59^ with Circos output^60^.

## Additional Information

**How to cite this article**: Makkonen, J. *et al*. Mitochondrial genomes and comparative genomics of *Aphanomyces astaci* and *Aphanomyces invadans. Sci. Rep.* **6**, 36089; doi: 10.1038/srep36089 (2016).

**Publisher’s note**: Springer Nature remains neutral with regard to jurisdictional claims in published maps and institutional affiliations.

## Figures and Tables

**Table 1:** Genetic content of *Aphanomyces astaci* and *A. invadans* mtDNA.

Respiratory chain proteins (ATP synthesis)
Complex I (*n *= 10)
*nad*1*, *nad*2*, nad*3*, nad4*, nad*4L*, nad5*, nad6*, nad7*, nad*9*, nad11
Complex III (*n *= 1)
cob*
Complex IV (*n *= 3)
*cox*1*, cox*2*, cox*3
Complex V (*n *= 4)
*atp*1*, atp*6*, atp*8*, atp*9*
**Ribosomal proteins**
Small subunit (*n *= 11)
*rps*2*, rps*3*, rps*4*, rps*7*, rps*8*, rps*10*, rps*11*, rps*12*, rps*13*, rps*14*, rps*19
Large subunit (*n *= 5)
*rpl2, rpl*5*, rpl*6*, rpl*14*, rpl*16
**Import proteins (*****n** *= **1)**
*sec*Y
**Translational RNAs**
Ribosomal RNAs (*n *= 2)
*rnn*S*(SSU), rnnL*(LSU)
tRNAs (*n *= 33)
*tRNA-*Gly_TCC_*, tRNA-Glu_TTC_*, *tRNA-*Asp_GTC_*, *tRNA-*Arg_TCT_*, *tRNA-*Ser_GCT_*, tRNA-Met_CAT_*, *tRNA-*
Pro_TGG_*, *tRNA-*Trp_CCA_*, *tRNA-*Trp_CCA_*, *tRNA-*Pro_TGG_*, tRNA-Met_CAT_*, tRNA-Ser_GCT_*, tRNA-
Arg_TCT_*, *tRNA-*Asp_GTC_*, *tRNA-*Glu_TTC_*, tRNA-Gly_TCC_*, *tRNA-*His_GTG_, *tRNA-*Val_TAC_, *tRNA-*Ile_GAT_,
*tRNA-*Gln_TTG_, *tRNA-*Arg_GCG_, *tRNA-*Phe_GAA_, *tRNA-*Ala_TGC_, *tRNA-*Lys_TTT_, *tRNA-*Met_CAT_, *tRNA-*Ser_TGA_,
*tRNA-*Cys_GCA_, *tRNA-*Leu_TAA_, *tRNA-*Leu_TAG_, *tRNA-*Met_CAT_, *tRNA-*Gly_GCC_, *tRNA-*Tyr_GTA_, *tRNA-*Asn_GTT_
**Unassigned open reading frames (*****n *****= 3)**
*ymf*98 (*orf*143)*, ymf*99 (*orf*217)*, ymf*101 (*orf*64)

The genes and tRNAs marked with an asterisk located in inverted repeat region.

**Table 2:** Mitochondrial genome size and number of putative open reading frames for a range of oomycete taxa with and without an inverted repeat.

Taxa	GenBank accession	Mt genome size (bp)	Inverted repeat size (bp)	Genome size, less 1 arm of inverted repeat	Number putative open reading frames
*Aphanomyces astaci*	KX405004	49,489	12,570	36,919	3
*Aphanomyces invadans*	KX405005	49,061	12,367	36,694	3
*Saprolegnia ferax*	AY534144	46,930	8,133	38,797	4
*Thraustotheca clavata*	NC022179	47,382	9,525	37,857	4
*Achyla hypogyna*	KF226724	46,840	7,973	38,867	5
*Pythium ultimum*	GU138662	59,689	21,950	37,737	7
*Pythium insidiosum*	AP014838	54,989	18,266	36,723	4
*Phytophthora ramorum*	DQ832718	39,314	1,150	38,164	9
*Phytophthora sojae*	DQ832717	42,977	—	42,977	13
*Phytophthora polonica*	KT946598	40,467	—	40,467	6
*Phytophthora andina*	HM590419	37,874	—	37,874	5
*Phytophthora ipomoeae*	HM590420	37,872	—	37,872	5
*Phytophthora mirabilis*	HM590421	37,779	—	37,779	5
*Phytophthora infestans*	AY898627	39,870	—	39,870	11
*Phytophthora phaseoli*	HM590418	37,914	—	37,914	5
*Peronospora tabacina*	NC028331	43,225	—	43,225	13
*Pseudoperonospora cubensis*	KT072718	38,553	—	38,553	6

Number of unassigned putative reading frames, counted only once when present in an inverted repeat.
